# How Environmental Knowledge Management Promotes Employee Green Behavior: An Empirical Study

**DOI:** 10.3390/ijerph18094738

**Published:** 2021-04-29

**Authors:** Wenyao Zhang, Ruzhi Xu, Yuan Jiang, Wei Zhang

**Affiliations:** 1 School of Finance, Qilu University of Technology (Shandong Academy of Sciences), Jinan 250535, China; wyzh@qlu.edu.cn (W.Z.); xrz@qlu.edu.cn (R.X.); 2 School of Management, Harbin Institute of Technology, Harbin 150010, China; yuanjiang@hit.edu.cn; 3 School of Public Administration, Sichuan University, Chengdu 610065, China

**Keywords:** employee green behavior, environmental knowledge sharing, environmental knowledge application, environmental behavioral intention, green perceived organizational support

## Abstract

As environmental protection has gradually become the focus of enterprises’ development, employee green behavior becomes an important and key antecedent to study this issue, but there have been less studies conducted with knowledge management. As a result, drawing on the theory of planned behavior and the organizational support theory, this study investigates how environmental knowledge practices (environmental knowledge sharing and environmental knowledge application) affect employee green behavior by using a questionnaire survey administered to 266 employees in China to reveal their complex relationship mechanism. The results show that environmental knowledge application and environmental knowledge sharing have a positive effect on employee green behavior; environmental behavioral intention mediates the relationship between environmental knowledge application and employee green behavior, and between environmental knowledge sharing and employee green behavior; green perceived organizational support positively moderates the relationship between environmental behavioral intention and employee green behavior. The findings shed new light on the development of employee green behavior literature and provide practical reference for strategies related to environmental protection for managers.

## 1. Introduction

As public concern about environmental protection has been growing in recent years [1,2], many organizations and institutes have raised awareness of environmental practices by adapting their businesses to take charge of appropriate natural resource management and environmental management [3,4]. However, most of them neglect to guide employees to perform green behavior but concentrate only on the improvement of technical changes. Undoubtedly, employee green behavior (EGB) exerts a potentially great impact on minimizing negative performance of activities in the workplace [5,6], because employees are treated as major assets in organizations [7] and their green behaviors are measurable individual behaviors that are helpful to achieve environmental sustainability in the workplace [8,9], which is similar to pro-environmental behavior (PEB) and environmentally sustainable behavior [10].

Existing scholars have studied EGB from different perspectives, such as green organizational climate [11,12], education [13,14], leadership [15], green human resource management (GHRM) practices [16], corporate social responsibility (CSR) [17,18], corporate environmental strategy [19,20,21], environmental servant leadership [22], and beliefs and attitudes [23,24]. For instance, Tian et al. (2020) investigated the relationship between EGB and pro-environmental attitude and how perception of green work climate affects EGB [24]. Thomas et al. (2017) stated that corporate environmental strategy positively influences green psychological climate and has a moderating effect on the relationship between green behavioral intention and next-day employee green behavior [21]. Richa (2019) aimed at revealing how GHRM practices foster employees’ environmentally responsible behaviors. Zhang et al. (2019) discussed how GHRM practices (training and education, rewards, employee life cycle, manager involvement, and employee empowerment) affect EGB in the workplace [20]. Mohammed et al. (2021) analyzed the link between CSR and EGB and the mediator impact of employees’ well-being on their link [18]. Other scholars have applied different theories to analyze EGB, of which the theory of planned behavior and organizational support theory are mostly used. For example, Rioux (2011) found there is a positive association between intention to act and battery collecting behavior based on TPB [25]. Nye and Hargreaves (2010) showed that drawing on the theory of planned behavior, subjective norm, attitude, and behavioral control positively influence acts of pro-environmental intention in the workplace [26]. Lamm et al. (2014) argued that perceived organizational support for environment embodies the beliefs of employees in terms of how their contributions to sustainability are valued by the organizations [27].

Further, from the study of Hines et al.’s (1987) classic meta-analysis, it was found that environmental knowledge is one most potent predictors of environmentally friendly behavior [28]. Environmental knowledge is defined as an individual’s ability to identify environmental concepts, signs, and behavior patterns [29]; revealing how they are responsible for the environment; and how environmental knowledge influences the environmental behavior of individuals [30]. Even in some cases, individuals with environmental knowledge are proven to become more concerned with environmental issues [31], and it is impossible for one individual to be conscious and care about environmental issues or act pro-environmentally if they know nothing about the environment [32]. As a result, environmental knowledge has been a major variable to explain pro-environmental behavior [29]. But how? What specific environmental knowledge behaviors could promote EGB, as how to deal with environmental issues and take the initiatives to engage in knowledge management rely upon sharing and applying the right knowledge at the right time to the right people [33]. Hence, this study reveals how environmental knowledge application and environmental knowledge sharing influence EGB. Meanwhile, in spite of the complexity of EGB, it is hard to be motivated by managers under traditional styles or approaches to leadership [34]. To further mobilize employee initiatives, there have been studies to analyze the direct effect of perceived organizational support for the environment (POS-E) [35]. Following that line of reasoning, drawing on the organizational support theory and theory of planned behavior, this study makes a comprehensive analysis to disclose the complex relationship mechanism among environmental knowledge application and sharing, environmental behavioral intention, green perceived organizational support, and EGB.

This article is organized as follows: The following section discusses the theoretical background and develops a theoretical model with a series of hypotheses, followed by methodology, data analysis, and results. Further, this article provides a discussion and conclusion in terms of key findings and implications for theory and practice.

## 2. Theoretical Background and Hypotheses

### 2.1. Theory of Planned Behavior

The theory of planned behavior has been extensively applied to explain human behavior in different contexts [36], holding the point that behavioral intention is influenced by attitude and shapes behavior in turn [37]. It argues an individual’s behavioral intention is capable of predicting their behavior well [31] and is thus understood as a strong internal stimulus for any behavior [38]. In this sense, it is one of the most reasonable models to explicate environmental behavior [39], where environmental behavioral intention reflects an individual’s disposition in environmental behavior [40] as it is an individual’s perceived subjective consciousness of environmental behavior. Drawing on this theory, this study analyzes how employees’ environmental knowledge sharing and environmental knowledge application affect EGB via their environmental behavioral intention.

### 2.2. Organizational Support Theory

**Figure 1:**
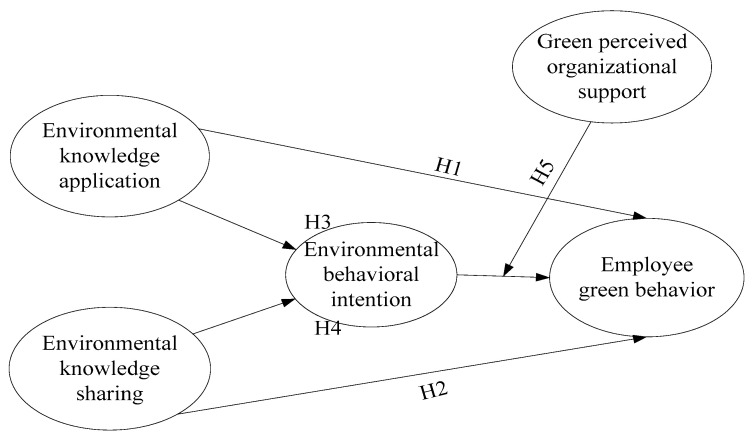
The conceptual model. Source: edited by authors.

Organizational support theory points out that employees care more about how they are treated in an organization, and thus, it provides a way to discern the degree to which organization values their contribution and is supportive to them [41]. It is evidenced that perceived organizational support has a positive effect on employees’ successful environmental initiatives, effort, and involvement in environmental management [42]. In this sense, green perceived organizational support (GPOS) refers to an employee’s perception of the degree to which their green contribution to the organization is valued. To this end, it is inferred that how employees are treated in an organization indicates the organization’s environmental orientation. Thus, this study also uses organizational support theory to illustrate how GPOS moderates the relationship between employee’s environmental behavioral intention and EGB, as shown in Figure 1.

### 2.3. Environmental Knowledge Application and Employee Green Behavior

As environmental knowledge greatly impacts EGB, environmental knowledge application refers to the utilization of environmental knowledge elements accumulated over time. Knowledge is a necessary way to overcome psychological barriers, such as misrepresentation, unconsciousness, or fear, even though knowledge is not taken as a reliable predictor of behavior any longer [40,43]. If employees have incorrect or no environmental knowledge, they will not make the right environmental choices [44,45]. In this sense, environmental knowledge application, as a power green lifter, is to increase employees’ initiatives to carry on green practices, which is conducive to correctly understanding and vitalizing employees’ positive subjective ideas of green practices while preventing their negative ideas of green practices.

In addition, the key to environmental knowledge application refers to how learned environmental knowledge is applied. It promotes the knowledge-recombinant capability to enable innovative performance based on a profound knowledge stock as knowledge fundamentals [46]. Further, employees’ stored environmental knowledge is taken as an intelligent tool to help identify good or bad green behavior, thus shaping their direction and scope of green activities, where EGB is a final form of expression of employees to use their environmental knowledge stored. It can thus be said that only when environmental knowledge is mastered and used by employees can it play a guiding role in green behavior. Hence, we propose the following hypothesis:

**Hypothesis** **1** **(H1).** *Environmental knowledge application has a positive effect on employee green behavior.*

### 2.4. Environmental Knowledge Sharing and Employee Green Behavior

Knowledge sharing refers to the exchange of information among employees in an organization [47]. It is deemed as the means by which employees make contribution to knowledge innovation and knowledge application in an organization [48]. Environmental knowledge sharing thus creates an organizational working atmosphere of environmental protection to facilitate employees’ willingness to communicate environmental knowledge, and thus, it increases employees’ cohesion to posse such environmental knowledge through subtly influencing them to generate more and better environmental knowledge and spread it from the individual experience level to the organizational level.

Additionally, environmental knowledge sharing enables the dissemination of environmental knowledge of employees who are more likely to generate awareness of green behavior. Prior studies indicate that knowledge value is increased during the process of knowledge sharing [49,50]. In this sense, environmental knowledge sharing encourages employees to generate more, new environmental knowledge and improve their original knowledge to a high level of quality, thereby positively directing employees’ tendency to perform green behavior. Thus, we propose the following hypothesis:

**Hypothesis** **2** **(H2).** *Environmental knowledge sharing has a positive effect on employee green behavior.*

### 2.5. Mediating Role of Environmental Behavioral Intention

Environmental behavioral intention is considered as an individual’s perceived subjective in taking part in environmental behavior, which implies his or her disposition in performing that environmental behavior [40]. Environmental knowledge application implies employees with environmental awareness possess a sense of duty or concern for environmental consequences. Through the effect of environmental behavioral intention, employees’ personal disposition and moral obligation are activated and they become passionate about environmental protection, thus, they are more willing to act with green behavior.

Specifically, employees’ environmental knowledge application presents an index to their consciousness and psychological sum of thinking and feeling about green behavior through behavioral intention, so that employees are more conscious of addressing environmental issues with related knowledge and hence, become willing to put environmental knowledge into practice. Employees’ environmental knowledge used refers to when they take the necessary green defenses to maintain their psychological green health and autonomously apply the environmental knowledge they have grasped to change unfavorable corporate environments and create a comfortable corporate environment for employees to survive and develop, embodying employees’ willingness to engage in green behaviors. Therefore, we propose the following hypothesis:

**Hypothesis** **3** **(H3).** *Environmental behavioral intention mediates the effect of environmental knowledge application on employee green behavior.*

Environmental knowledge sharing is taken as a whole concept that directly creates a kind of organizational climate under which employees highly trust each other, so it is easy to freely circulate knowledge, and is conductive to increasing effective cooperation and minimizing value incongruence [47]. It is instrumental in expanding environment-related knowledge and experience to lead to successful EGB by generating a consistent tendency of environmental behavioral intention and thus, standardizing a series of employees’ green behaviors.

In addition, environmental knowledge sharing in the organization shows an environmental cognitive schema to display organizational members’ common perceptions and attitudes towards the environment [32]. Intention, as a direct behavior predictor, plays a substantial role in the relationship between attitude and pro-environmental behavior [39]. Environmental knowledge sharing reflects a positive attitude towards green environment within the organization, indicating that employees’ intuitive manifestation of their environment moves via environmental behavioral intention to express their personal inclination, making them more motivated to get involved in pro-environmental actions [51]. Thus, we propose the following hypothesis:

**Hypothesis** **4** **(H4).** *Environmental behavioral intention mediates the effect of environmental knowledge sharing on employee green behavior.*

### 2.6. Moderating Role of Green Perceived Organizational Support

An individual’s behavior is impacted by the organization they are part of [52]. According to organizational support literature, employees’ perceived organizational support for their efforts put into environment tends to satisfy their tasks or jobs [53]. How employees are treated in the organization represents their organization’s environmental orientation. When employees’ GPOS is increased, their expectations and positive intentions are improved because they are aware that the organization supports them to be active and environmentally compatible with organizations towards environmental behaviors, which will lift their behavioral intention to perform green behavior.

Further, GPOS refers to employees’ beliefs with regard to how much the organization is concerned with environmental values in the workplace [29]. It is deemed as an incentive critical for employees to act in a pro-environmental manner [31]. Under such circumstances, employees’ beliefs tend to be congruent with environmental climate, goal, and values within the organization. The more GPOS employees have a sense of, the more likely it entails stronger consistent intention to achieve organizational aspirations of EGB. To this end, GPOS prompts the effect of environmental behavioral intention on EGB. Therefore, we propose the following hypothesis:

**Hypothesis** **5** **(H5).** *Green perceived organizational support positively moderates the relationship between environmental behavioral intention and employee green behavior.*

## 3. Methods

### 3.1. Sample

As the relationship between economic development and environmental protection is studied deeply, the ecological career of environmental protection has achieved solid results in China, starting from the decision to improve the ecological environment to achieving high-quality economic green development, since China began to implement the most stringent New Environmental Protection Law in history and crack down on environmental violations of enterprises with uncapped punishment in 2015. Hence, to empirically test the theoretical model, this study selected samples in China to conduct a cross-sectional field survey. Taking the concept of green behavior into account, this study considered companies that have achieved good green behavior effect in environmental protection by employing low-carbon and green production processes and transportation as green samples, involving manufacturing, food, cosmetics, and energy conservation and environmental protection industries, during a 4-month period in 2020.

We divided the questionnaires into two parts and sent them to the participants two times in order to decrease common method variance. The survey at Time 1 consisted of their demographic background and independent variables, namely, environmental knowledge application, environmental knowledge sharing, environmental behavioral intention, and green perceived organizational support. The survey was sent to collect participants’ responses about the dependent variable, employee green behavior, about half a month later at Time 2. A total of 313 survey questionnaires were administered to employees, managers, and executives in 57 companies engaged in green behavior and environmental protection in Wuhan, Shanghai, Jinan, and Harbin. The questionnaires returned with incomplete or invalid answers were removed. A total of 266 valid questionnaires were collected for data analysis. Table 1 reports demographic profile of respondents.

### 3.2. Measures

The questionnaire survey was developed in two parts to test the hypotheses. Further, the approach of translation and back-translation procedures [54] was used to deal with the translation between all English and Chinese items. The instrument consisted of 22 measurements to define five constructs, most of which were adapted from the existing literature. The seven-point Likert scale was utilized by measuring all items corresponding to constructs, and the choices of answers ranged from ‘‘disagree strongly” (1) to ‘‘agree strongly” (7).

The scale items for green perceived organizational support were adapted from Eisenberger at al. (1986) [39], from De Roeck and Farooq (2017) for employee green behavior [55], from Bock et al. (2005) [56] and Tabernero and Hernández (2011) for environmental behavioral intention [54], and from Connelly et al. (2012) for environmental knowledge sharing [57]. The scale items for environmental knowledge application were developed from Gold et al. (2001) [58] and Egena and Rajenthyran (2020) [59] to cater to this study, and five items strongly associated were revised and selected. Table 2 shows items for variables.

The researchers controlled for employees’ gender, tenure, and education level, and emphasized the voluntary nature of all participants. Especially, we selected employees with high education levels at a proper age or who worked in the same company for a longer period, because all this may better articulate how they felt about the company they have been working in, which may in turn influence their judgement and perception of their knowledge behavior and its resulting impact on their green behavior. Moreover, the researchers were not biased in favor of either gender, in a way to ensure gender balance for the voluntary participants selected. To this end, it was objective and significant to interpret their green behaviors.

### 3.3. Data Reliability and Validity

The reliability was assessed by Cronbach’s α, as reported in Table 2, which all exceeded the acceptable level of 0.7. As a result, it was concluded the questionnaires were reliable and appropriate for use. The Kaiser–Meyer–Olkin (KMO) value was 0.903, implying that the sample size was sufficient to satisfy the restrictive conditions of sample factor analysis. According to Bartlett spherical inspection, the Chi-square value was 3593.497 (*p* < 0.001), showing that all items could be correlated and the common factors extracted. Exploratory factor analysis (EFA) was performed by employing the maximum variance positive rotation, as shown in Table 3. A total of 22 items were loaded to five common factors, whose variance contribution reached up to 70.91%. Every factor item corresponded to the appropriate underlying construct. The factor loadings were between 0.666 and 0.826, implying latent variables achieved convergent and discriminant validity.

## 4. Results

Table 4 illustrates the descriptive statistics of all variables in this study. The average scores were 3.48, 3.37,3.61, 3.79, and 3.50 for environmental knowledge application (EKA), environmental knowledge sharing (EKS), environmental behavioral intention (EBI), green perceived organizational support (GPOS), and employee green behavior (EGB), respectively. Our results indicated that environmental knowledge application (*r* = 0.46, *p* < 0.01), environmental knowledge sharing (*r* = 0.44, *p* < 0.01), environmental behavioral intention (*r* = 0.42, *p* < 0.01), and green perceived organizational support (*r* = 0.30, *p* < 0.01) were positively correlated with employee green behavior. Environmental knowledge application was positively correlated with environmental knowledge sharing (*r* = 0.58, *p* < 0.01) and environmental behavioral intention (*r* = 0.48, *p* < 0.01).

We adopted a three-step procedure to test the hypotheses. First, in order to test whether the variables can be distinguished from each other, we conducted a confirmatory factor analysis (CFA). The results showed that the five-factor model (environmental knowledge application, environmental knowledge sharing, environmental behavioral intention, employee green behavior, and green perceived organizational support) fitted the data well (χ^2^ = 333.02, df = 199, RMSEA = 0.05, CFI = 0.96, SRMR = 0.04), which was better than all other models (279.33 ≤ Δχ^2^ ≤ 1379.63, *p*s < 0.001), indicating that the variables had good discriminative validity and supported the measurement model.

**Figure 2:**
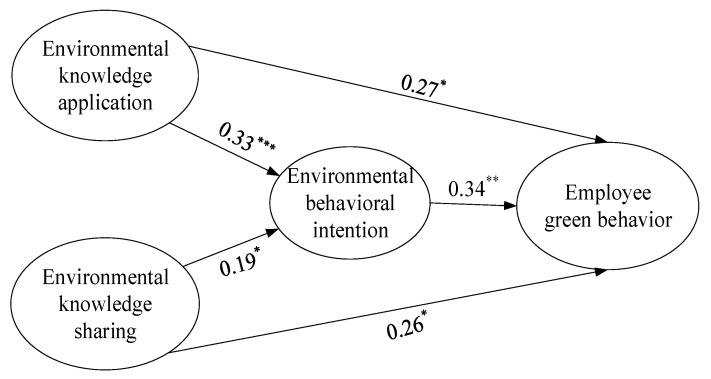
Structural equation model results of primary and mediating effects. Source: edited by authors. Notes: * *p* < 0.05, ** p < 0.01, *** *p* < 0.001.

Second, by incorporating environmental knowledge application, environmental knowledge sharing, environmental behavioral intention, and employee green behavior into the analysis, we tested the impact of environmental knowledge application and environmental knowledge sharing on employee green behavior and environmental behavioral intention, as well as the mediator effect of environmental behavioral intention, with the results shown in Figure 2. According to the analysis results, the structural equation model had a strong goodness of fit (χ^2^ = 306.57, df = 177, RMSEA = 0.05, CFI = 0.96, SRMR = 0.05). In addition, environmental knowledge application and environmental knowledge sharing were significantly positively correlated with employee green behavior (β1 = 0.27, *p*1 < 0.05; β2 = 0.26, *p*2 < 0.05, respectively); thus, Hypothesis 1 and Hypothesis 2 were supported.

Environmental knowledge application and environmental knowledge sharing were significantly positively correlated with employee green behavior (β1 = 0.33, *p*1 < 0.001; β2 = 0.19, *p*2 < 0.05); employees’ environmental behavioral intention is significantly positively correlated with employee green behavior (β = 0.34, *p* < 0.01). By utilizing the bootstrap approach to test the mediating effect, the results implied that environmental behavioral intention significantly mediated the impact of environmental knowledge application on employee green behavior (effect size = 0.11, 95% CI = [0.03, 0.24]) and the impact of environmental knowledge sharing on employee green behavior (effect size = 0.06, 95% CI = [0.01, 0.16]); hence, Hypothesis 3 and Hypothesis 4 were supported. In the effect of environmental knowledge application on employee green behavior, 29% can be explained by environmental behavioral intention. In the effect of environmental knowledge sharing on employee green behavior, 20% can be explained by environmental behavioral intention. According to Hadi et al. (2016), it is partial mediation when it is 20–80%.

**Figure 3:**
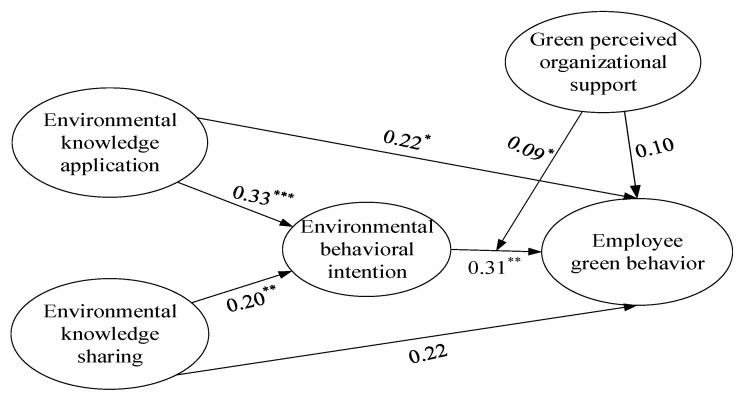
Structural equation model results of moderating effect. Notes: * *p* < 0.05, ** *p* < 0.01, *** *p* < 0.001.

Finally, we incorporated green perceived organizational support into the analysis, and introduced the single factor product index method to test the moderating effect, with results shown in Figure 3. Results show that the structural equation model has strong goodness of fit (χ2 = 433.73, df = 281, RMSEA = 0.05, CFI = 0.96, SRMR = 0.05), the interactive impact environmental behavioral intention and green perceived organizational support was positive and significant (β = 0.09, *p* < 0.05). The simple slope test results indicated that when green perceived organizational support was high (+SD), environmental behavioral intention was significantly positively correlated with employee green behavior (β = 0.47, *p* < 0.01); when green perceived organizational support was low (−SD), the relationship between environmental behavioral intention and employee green behavior was not significant (β = 0.16, n.s.). Hence, Hypothesis 5 was supported.

## 5. Discussion

This study examines the complex relationship among variables to examine their roles in driving EGB. Specifically, environmental knowledge is defined as a tool to denote awareness and knowledge regarding environmental issues and solutions [60]. Employees’ environmental knowledge application presents their interpretation of environmental information by reference to previous experience, values, and insight on the environment, and manifests how employees use environmental knowledge in actual activities, thus directly impacting their green behavior. In other words, environmental knowledge application brings about a positive rise in environmental awareness through changing employees’ pro-environmental behavior, increases their recognition of environmental problems and causes, and directs their propensity to engage in green behavior by dominating their environmental behavioral intention. Therefore, H1 and H3 are supported.

Environmental knowledge shared makes employees understand the harm caused by environmental pollution that may threaten their lives and corporate development, so their awareness of environmental risks are improved and their pro-environmental behavioral intention is further impacted positively [61]. In this sense, H2 is verified. Additionally, the act of environmental knowledge sharing displays an organization’s supportive attitude oriented by environmental values, where it entails to raise employees’ beliefs that the organization supports their green behavior and practices [3], thus generating environmental behavioral intention to facilitate employee green behavior. Thus, H4 is verified.

Based on the theory of planned behavior, it is reasonable to suggest that environmental intention affects environmental behavior [62], but in our context, the relationship between them is subject to green perceived organizational support. The reason may be explained by the fact that the organizational support theory believes a social exchange relationship is formed between employees and their organization, which also conforms to the essence of reciprocity norm [63]. When employees are motivated and supported to take reciprocated actions, it will show their greater affective commitment to the organization and perform citizenship behavior [64]. In other words, green perceived organizational support makes employees feel cared, recognized, and respected in a way to meet their social and emotional needs, so as to promote their environmental behavioral intention to cooperate and realize green value consistency with the organization. Thus, H5 is supported.

### 5.1. Theoretical Implications

This study has several theoretical implications. First, it enriches the theoretical literature related to employee green behavior by providing empirical evidence to study how knowledge management practices of employees influence their green behavior [65], which contributes to the ongoing debate of whether environmental knowledge affects pro-environmental behavior (green behavior) [66]. The results of Pihui et al. (2020) [29] show a positive relevance between environmental knowledge management practices and employee green behaviors. Our results are opposite to the existing finding that knowledge is no longer a reliable behavior predictor [41], demonstrating that environmental knowledge created by knowledge behavior has a positive effect on employee green behavior.

Second, our study contributes to the employee green behavior literature by empirically establishing a holistic theoretical framework with environmental behavioral intention as a mediator and with green perceived organizational support as a moderator to verify the studies of Casalo and Escario (2018) [67] and Lee et al. (2015) [68] that showed other probable intermediaries exist in the causal relationship between environmental knowledge and environmentally friendly action. Our findings also complement the deficiency of environmental behavior studies by exploring employee green behavior in the workplace in a Chinese context, as there have been a stream of researches conducted on pro-environmental behavior in other developed countries rather than in the context of China [69,70,71].

Third, this study extends the employee green behavior literature by integrating theory of planned behavior and organizational support theory to investigate a direct relationship between environmental behavioral intention and employee green behavior, which extends the conclusion of Milfont and Duckitt’s study [62] that intention has an indirect impact on the prediction of behavior. Further, this study extends the current research development of focusing on individual-level psychological explanations to analyze the relationship between pro-environmental intention and pro-environmental behavior based on the theory of planned behavior and organizational support theory [72].

### 5.2. Practical Implications

Drawing insight from our theoretical hypotheses, our findings provide practical strategy references for Chinese or foreign companies that actively carry out environmental or green behaviors. First of all, environmental knowledge sharing and environmental knowledge application can promote employee green behavior and hence, companies should focus on setting up diversified activities to stimulate knowledge sharing and application opportunities among employees, thereby facilitating employee green behavior.

Secondly, environmental behavioral intention mediates the relationship between environmental knowledge sharing and employee green behavior and between environmental knowledge application and employee green behavior. These findings help companies enhance employees’ awareness of and attitude towards environmental knowledge through environmental knowledge sharing and application, strengthen their understanding of green behavior, and supplement environmental knowledge in the sharing process. This creates an atmosphere for sharing environmental knowledge between enterprises, allowing employees to clearly understand the importance of environmental knowledge, which enables them to be psychologically compatible with the company’s green development intention, and is helpful to forming environmental behavioral intention and then green behavior.

Thirdly, green perceived organizational support moderates the relationship between environmental behavioral intention and employee green behavior. The results demonstrate that managers should clearly support and encourage green behaviors within the company, so that employees can perceive the company’s support for their environmental contributions from engaging in green behaviors and make them generate green beliefs. This can be realized by managers taking different supporting measures, such as green behavior appraisal activities and incentive measures. By perceiving strong, green organizational support, employees’ environmental behavioral intention has a higher impact on their green behavior.

## 6. Conclusions

Drawing from the organizational support theory and theory of planned behavior, this study reveals the relationship mechanism between employees’ knowledge management practices and employee green behavior with green perceived organizational support as a moderator and environmental behavioral intention as a mediator. The results show that environmental knowledge application and environmental knowledge sharing have a positive effect on employee green behavior; environmental behavioral intention mediates the relationship between environmental knowledge application and employee green behavior and between environmental knowledge sharing and employee green behavior; and green perceived organizational support positively moderates the relationship between environmental behavioral intention and employee green behavior. This study expands the employee green behavior literature and empirically contributes to research on the relationship between knowledge management practices and green behavior by building an integrated model and applying planned behavior and organizational support theories to employee green behavior research.

This study has some limitations. First, this empirical study only selects 266 samples, so future studies are suggested to test our theoretical hypotheses with a larger sample size or qualitative methods (longitudinal design and multisource data) to reinforce our results with triangle validation. Second, the proposed conceptual model considered the effect of only three components (environmental knowledge sharing, environmental knowledge application, and green perceived organizational support) on employees’ environmental behavioral intention and green behavior. Thus, future work should consider including other knowledge-related or organizational factors by exploring the deep inner attributes of knowledge activities and their connections to employee green behavior within organizations.

## Figures and Tables

**Table 1:** Demographic profile of respondents.

Measure	Items	Frequency	Percentage %
Gender	Male	112	42.1
	Female	154	57.9
Education level	Junior college or below	17	6.4
	Bachelor	157	59
	Master	55	20.7
	Doctor	37	13.9
Tenure: How long have you been working in this company?	1 to 5 years	179	67.3
	5 to 10 years	54	20.3
	10 to 15 years	25	9.4
	15 to 20 years	2	8
	20 years or above	6	2.3

Source: edited by authors.

**Table 2:** Items for variables.

Variable	Item	Cronbach’s α
Green perceived organizational support (GPOS)	GPOS1: The organization values my contribution to environmental management.	0.865
	GPOS2: The organization really cares about my environmental goals and values.	
	GPOS3: The organization cares about my opinions on sustainability.	
	GPOS4: The organization takes pride in my accomplishments on environmental issues at work.	
Employee green behavior (EGB)	EGB1: I adequately complete assigned duties in environmentally friendly ways.	0.889
	EGB2: I fulfill responsibilities specified in my job description in environmentally friendly ways.	
	EGB3: I perform job tasks that are expected from me in environmentally friendly ways.	
	EGB4: I take a chance to get actively involved in environmental protection at work.	
	EGB5: I take initiatives to act in environmentally friendly ways at work.	
Environmental behavioral intention (EBI)	EBI1: I would give part of my income if I were certain that the money would be used to prevent environmental pollution.	0.860
	EBI2: I would agree to an increase in taxes if the extra money were used to prevent environmental pollution.	
	EBI3: The government should reduce environmental pollution, but it should not cost me any money.	
Environmental knowledge application (EKA)	EKA1: I take advantage of new environmental knowledge.	0.861
	EKA2: I use environmental knowledge to improve efficiency.	
	EKA3: I make environmental knowledge accessible to those who need it.	
	EKA4: I quickly link sources of environmental knowledge to solving problems.	
	EKA5: I have processes for applying environmental knowledge learned from mistakes.	
Environmental knowledge sharing (EKS)	EKS1: This coworker looks into my environmental requests to make sure their answers are accurate.	0.887
	EKS2: This coworker explains everything about environment very thoroughly.	
	EKS3: This coworker answers all my environmental questions immediately.	
	EKS4: This coworker tells me exactly what I need to know about environment.	
	EKS5: This coworker goes out of their way to ensure that they understand my environmental requests before responding.	

Source: edited by authors.

**Table 3:** Exploratory factor analysis results.

	1	2	3	4	5
EKS1	0.154	0.708	0.274	0.169	0.169
EKS2	0.119	0.802	0.266	0.174	0.115
EKS3	0.176	0.729	0.246	0.181	0.123
EKS4	0.261	0.720	0.162	0.206	0.147
EKS5	0.125	0.814	0.085	0.081	0.108
EKA1	0.211	0.357	0.720	0.165	0.241
EKA2	0.151	0.015	0.751	−0.002	0.030
EKA3	0.210	0.299	0.761	0.094	0.253
EKA4	0.237	0.312	0.708	0.114	0.219
EKA5	0.042	0.211	0.666	0.139	0.057
EGB1	0.800	0.133	0.213	0.068	0.160
EGB2	0.785	0.104	0.173	0.092	0.065
EGB3	0.800	0.182	0.143	0.062	0.053
EGB4	0.801	0.176	0.060	0.130	0.093
EGB5	0.761	0.148	0.126	0.096	0.293
EBI1	0.218	0.094	0.220	0.175	0.788
EBI2	0.175	0.157	0.233	0.137	0.845
EBI3	0.139	0.270	0.074	0.121	0.804
GPOS1	0.111	0.170	0.125	0.818	0.127
GPOS2	0.088	0.154	0.094	0.826	0.156
GPOS3	0.117	0.125	0.069	0.784	0.006
GPOS4	0.061	0.158	0.075	0.819	0.145

Extraction method: principal component analysis. Rotation method: Caesar normalized maximum variance method. A rotation has converged after six iterations. Source: edited by authors.

**Table 4:** Descriptive statistics of all variables.

	Mean	S.D.	Tenure	Education	EKA	EKS	EBI	GPOS	EGB
Tenure	2.79	1.21	1						
Education	2.42	0.86	0.37 **	1					
EKA	3.48	1.42	0.19 **	0.19 *	1				
EKS	3.37	1.47	0.22 **	0.09	0.58 **	1			
EBI	3.61	1.30	0.15 *	1.42 *	0.48 **	0.45 **	1		
GPOS	3.79	1.68	0.05	−0.01	0.32 **	0.42 **	0.36 **	1	
EGB	3.50	1.70	0.25 **	0.27 **	0.46 **	0.44 **	0.42 **	0.30 **	1

Notes: * *p* < 0.05, ** *p* < 0.01.

## Data Availability

The data presented in this study are available on request from the corresponding author. The data are not publicly available due to privacy reasons.
